# Supplementary material for: Combining lipoic acid to methylene blue reduces the Warburg effect in CHO cells: From TCA cycle activation to enhancing monoclonal antibody production
Source: PLoS One. 2020 Apr 16;15(4):e0231770. doi: 10.1371/journal.pone.0231770 (PMC7162497; doi:10.1371/journal.pone.0231770)
Supplement: S1 Fig — Specific consumption and production rates of glucose (A), lactate (B), glutamine (C) and glutamate (D) were measured in the extracellular medium for the various drug treatments. Glycolytic specific rates qGLC and qLAC were calculated on 0–48 h and 48–120 h based on the metabolic shift observed at 48 h. Glutaminolytic rates qGLN and qGLU were calculated before (0–72 h) and after (72–120 h) glutamine depletion. All conditions were statistically compared to the control by one-way ANOVA. (DOCX) [file pone.0231770.s001.docx]

**

**

**Fig S1. Specific consumption and production rates**

Specific consumption and production rates of glucose (A), lactate (B), glutamine (C) and glutamate (D) were measured in the extracellular medium for the various drug treatments. Glycolytic specific rates q_GLC_ and q_LAC_ were calculated on 0-48 h and 48-120 h based on the metabolic shift observed at 48 h. Glutaminolytic rates q_GLN_ and q_GLU_ were calculated before (0-72 h) and after (72-120 h) glutamine depletion. All conditions were statistically compared to the control by one-way ANOVA.
