# Supplementary material for: Combining lipoic acid to methylene blue reduces the Warburg effect in CHO cells: From TCA cycle activation to enhancing monoclonal antibody production
Source: PLoS One. 2020 Apr 16;15(4):e0231770. doi: 10.1371/journal.pone.0231770 (PMC7162497; doi:10.1371/journal.pone.0231770)
Supplement: Attachment — Submitted filename: Response to Reviewers Comments.docx [file pone.0231770.s003.docx]

*We would like to thank the Associate editor as well as the reviewers for their helpful and valuable comments.*

Editor Comments (if provided):

There are several areas that can be revised to improve the manuscript. First, lactate levels in the culture supernatants are needed to support the notion that the treatments reduce lactate production and push pyruvate into TCA cycle.

- We agree, and Figure 2 showing metabolites concentration in culture media has been provided. A clear difference is visible only in the 100 µM ALA condition, however it is understood that specific values, accounting for the cell concentration, are more representative than these raw concentrations.

Second, methylene blue enhanced mAB production, but from the data presented, lipoic acid did not enhance mAB production. This is not consistent with the claim in the title.

- We agree and the manuscript title has been modified to “Combining lipoic acid to methylene blue reduces the Warburg effect in CHO cells: from TCA cycle activation to enhancing monoclonal antibody production”.

Third, methylene blue and lipoic acid both reduce Warburg effects, why are they different in effects on mAB productions? Any other off-targets effects of the compounds?

- These questions are crucial to the understanding of our work, and precisions are already included in the manuscript:
  - Line 344 "Indeed, α-LA is known to activate multiple entry-point enzymes to the TCA cycle [56]."
  - Lines 363-368: "The mitochondrial activity and redox balance are strongly dependent on α-LA. It does not only have antioxidant properties but it also acts as cofactor of many mitochondrial enzymes in addition to its action on PDH [57]. For instance, the regulation of complex I production of superoxide anion through its interaction with 2-oxoglutarate dehydrogenase [56] can account in part for the restriction in ROS production (Fig 4)."
  - Specifically concerning MB, we modified line 387: “MB is a potent redox exchanger acting as an electron shuttle in the mitochondria, bypassing complexes I to III of the ETC and resulting in decreased ROS production [46, 58].”

N.B. In supplement, Prof. Mario Jolicoeur ORCID number has been indicated: [https://orcid.org/0000-0002-0875-7265](https://orcid.org/0000-0002-0875-7265?lang=en)

Reviewer #1: This is a very solid study – from background layout, to research strategy, experiment design, data collection and result presentations are all good. Results are convincing and supporting the conclusions. The mechanisms on these drugs alpha-LA and MB were also studied. I only have two comments:

(1) In addition to measure mAb titers by ELISA, the quality and integrity of assembled IgG could be tested by SDS-PAGE after IgG purification.

- We agree with the reviewer that mAb quality is central when addressing a bioprocess performance. However, the aim of this study was primarily to test the effect of drugs that are used in human to modulate cell energetics, can be useful to reduce the Warburg effect, which is known to limit mAb productivity in CHO cells. In order to clarify this point, we have added these sentences in the Conclusion from line 418 : “At the molecular level, metabolic changes can impact mAb quality, i.e. the glycosylation profile and biological activity. Further dedicated studies would be required to identify optimal lipoic acid and methylene blue concentrations and ratios to preserve the mAb molecular properties. We chose to focus on the net production of antibody as it reflects the general metabolic state of the cell. Using this criterium, we showed that, even more than the imbalance between glycolysis and respiration, the mitochondrial capacity was critical for productivity in this CHO cell line.”

  (2) As the IgG yields also depend on the particular mAb clone used in general. I wonder whether the drug treatment approaches studied here can be applied to different mAb clones?
- We agree with the reviewer, in addition to the CHO cell line effect. It is expected that each production platform (CHO cell line – mAb expression system) will present variations of performance when assessing culture management strategies. One unexpected example is precisely the no-effect of DCA on the mAb productivity of CHOs studied in this study, conversely to an enhancement reported in other CHOs used in the study of Buchsteiner et al. 2018. Further works aiming at identifying optimal drugs’ respective concentrations and ratios on mAb productivity will definitely require scanning various CHO cell lines harboring different recombinant expression systems.
